# Photoprotective effects of *Sargassum thunbergii* on ultraviolet B-induced mouse L929 fibroblasts and zebrafish

**DOI:** 10.1186/s12906-022-03609-x

**Published:** 2022-05-21

**Authors:** Bei Chen, Honghong Chen, Haidong Qu, Kun Qiao, Min Xu, Jingna Wu, Yongchang Su, Yan Shi, Zhiyu Liu, Qin Wang

**Affiliations:** 1 Fisheries Research Institute of Fujian, Key Laboratory of Cultivation and High-value Utilization of Marine Organisms in Fujian Province, No. 7, Haishan Road, Huli District, Xiamen, 361013 Fujian China; 2 School of Life Sciences, Xiamen University, South Xiangan Road, Xiang’an District, Xiamen, 361102 Fujian China; 3 School of Environmental Science and Engineering, Southern University of Science and Technology, Shenzhen, 518055 Guangdong China; 4 College of the Environment and Ecology, Xiamen University, Xiamen, 361102 Fujian China; 5 Xiamen Medical College, Xiamen, 361023 Fujian China

**Keywords:** *Sargassum thunbergii*, Ultraviolet B, Photoprotective, L929 fibroblast, Zebrafish

## Abstract

**Background:**

Chronic exposure to ultraviolet B (UVB) causes a series of adverse skin reactions, such as erythema, sunburn, photoaging, and cancer, by altering signaling pathways related to inflammation, oxidative stress, and DNA damage. Marine algae have abundant amounts and varieties of bioactive compounds that possess antioxidant and anti-inflammatory properties. Thus, the objective of this study was to investigate the photoprotective effects of an ethanol extract of *Sargassum thunbergii*.

**Methods:**

*Sargassum thunbergii* phenolic-rich extract (STPE) was prepared, and its activity against UVB damage was evaluated using L929 fibroblast cells and zebrafish. STPE was extracted and purified by 40% ethanol and macroporous resin XDA-7. Reactive oxygen species (ROS) and antioxidant markers, such as superoxide dismutase (SOD), catalase (CAT) activities, and malondialdehyde (MDA) content were analyzed. The effect of STPE on UVB-induced inflammation was determined by inflammatory cytokine gene and protein expression. The expression of signaling molecules in the Nuclear Factor KappaB (NF-κB) pathway was determined by western blotting. DNA condensation was analyzed and visualized by Hoechst 33342 staining. In vivo evaluation was performed by tail fin area and ROS measurement using the zebrafish model.

**Results:**

The total polyphenol content of STPE was 72%. STPE reduced ROS content in L929 cells, improved SOD and CAT activities, and significantly reduced MDA content, thereby effectively alleviating UVB radiation-induced oxidative damage. STPE inhibited the mRNA and protein expression of TNF-α, IL-6, and IL-1α. STPE reversed DNA condensation at concentrations of 20 and 40 μg/mL compared with the UVB control. Moreover, STPE inhibited NF-κB signaling pathway activation and alleviated DNA agglutination in L929 cells after UVB irradiation. Additionally, 1.67 μg/mL STPE significantly increased the tail fin area in zebrafish, and 0.8–1.6 μg/mL STPE effectively eliminated excessive ROS after UVB radiation.

**Conclusions:**

STPE inhibited UVB-induced oxidative stress, inflammatory cytokine expression, and DNA condensation via the downregulation of the NF-κB signaling pathway, suggesting that it prevents UVB-induced photodamage, and has potential for clinical development for skin disease treatment.

**Supplementary Information:**

The online version contains supplementary material available at 10.1186/s12906-022-03609-x.

## Background

Among the types of ultraviolet (UV) rays that are not absorbed by the ozone layer, ultraviolet B (UVB) (wavelength 280–315 nm) is a major environmental risk factor for the pathogenesis of skin damage [1]. UVB delivers more energy into the skin than UVA (320–400 nm). Many skin diseases, such as cancer, photoaging, sunburn, and pigmentation, are closely associated with UVB exposure. Owing to its short wavelength, UVB is mainly absorbed in the epidermis; however, it can also penetrate this skin layer to reach the dermis and affect the physiological functions of fibroblasts [2]. Studies on UVB-irradiated skin or dermal fibroblasts have mainly focused on DNA mutations [3], inflammation [4], and photoaging [1, 5].

Marine phenolic compounds exhibit a wide range of physiological properties, including antioxidant, anti-inflammatory, anti-tumor, and anti-microbial properties [6, 7]. Recently, the UV protection activity of marine polyphenols has been intensively focused upon owing to the growing demand for anti-aging, anti-allergic, and whitening natural products in the skin health industry. Fucodiphlorethol G extracted from *Ecklonia cava* increases the viability of human keratinized cells exposed to UVB radiation by absorbing UVB, removing intracellular reactive oxygen species (ROS), and reducing 8-isoprostane generation and DNA fragmentation [8]. The phlorotannin fucofuroeckol-A, derived from *Ecklonia stolonifera*, demonstrates protective effects against UVB-induced allergic reactions in RBL-2H3 mast cells by decreasing histamine release, increasing intracellular Ca^2+^, inhibiting IL-1β and TNF-α production, and scavenging ROS [9]. Diphlorethohydroxycarmalol extracted from *Ishige okamurae* exhibits strong protective properties against UVB radiation via reducing intracellular ROS levels, reducing damaged DNA tail length, and causing morphological changes in fibroblasts [10].

In the last decades, marine algae have been an important source of natural products to find new compounds with pharmacological and nutraceutical properties. *Sargassum thunbergii,* an economically important brown algae located on China and Japan seas, contains many bioactive compounds, such as indole derivatives, indolediketopiperazine alkaloids, quinone derivatives, polyphenols, and polysaccharides [11]. *S. thunbergii* lives in the intertidal zone undergoing multiple changes due to tidal changes, including high solar irradiances, desiccation, changes in salinity, and temperature. Therefore, we speculated that phenolic compounds from *S. thunbergii* might possess UV protective activity. However, to our knowledge, the photoprotective activity of *S. thunbergii* was unreported previously.

The mouse L929 fibroblast cell line is reportedly an effective in vitro screening test system for the phototoxicity assessment of drugs/chemicals [12]. Zebrafish (*Danio rerio*) are increasingly being considered as a model system for evaluating the efficacy of UVB protective compounds against fin damage and ROS generation [13, 14]. In this study, we established a UVB-exposed L929 cell and zebrafish photodamage model to study the UVB photoprotective activity of *S. thunbergii* phenolic-rich extract (STPE).

## Methods

### Reagents

A PierceTM BCA Protein Assay Kit, RPMI1640 medium, DPBS/MODIFIED, Trypsin-EDTA Solution, fetal bovine serum, and dual antibodies were purchased from Thermo Fisher Scientific (Waltham, MA, USA). A CellTiter 96 Aqueous Non-Radioactive Cell Proliferation Assay (MTS) kit was obtained from Promega (Madison, WI, USA) and phenazine methosulfate was purchased from Sigma-Aldrich (St. Louis, MO, USA). High Efficiency RIPA tissue/cell rapid lysis solution was acquired from Beijing Solarbio Science & Technology Co., Ltd. (Beijing, China). All antibodies were purchased from Abcam (Cambridge, MA, USA) or Cell Signaling Technology (Beverly, MA, USA) as follows: Nuclear Factor KappaB (NF-κB) p65 (D14E12) XP Rabbit mAb (#8242, dilution 1/1000), p-NF-κB p65 (Ser536) (93H1) rabbit mAb (#3033, dilution 1/1000), anti-IκBα antibody (ab32518, dilution 1/1000), anti-SDHA antibody (ab137040, dilution 1/1000), and anti-Lamin B antibody (ab133741, dilution 1/1000).

### Phenolic compound extraction and purification

The material used in the preparation of the *S. thunbergii* phenolic-rich extract was obtained from the coastal reefs in Dongtou Island, Zhejiang Province, China. The algal handling procedures in this study comply with local and national regulations. The Committee for Conservation of Aquatic Germplasm, from the Mariculture Research Institute of Zhejiang, China, approved these experiments. The wild type *S. thunbergii* algae was identified by Fujian Marine and Fisheries Judicial Expertise Center. A voucher specimen (ST20190402) was deposited at the Mariculture Research Institute of Zhejiang, China. Fresh *S. thunbergii* was delivered to Xiamen via cold chain logistics, followed by washing and freeze-drying. Dried *S. thunbergii* was crushed and powdered using a grinding mill. After 100-mesh screening, the powder was immersed in 40% (v/v) ethanol-water solution (solid to liquid ratio 1:15) for 60 min at 70 °C. After a double extraction, the extract was filtered using a Buchner funnel under vacuum. The supernatants were centrifuged at 12000 rpm for 30 min and concentrated to remove ethanol using a rotary evaporator (Buchi, Flawil, Switzerland). The crude *S. thunbergii* extract was further purified to obtain the STPE.

To purify the STPE, an XDA-7 macroporous resin (Sunresin Park, Xi’an, China) was used. Prior to use, the resin was activated by soaking with 95% ethanol for 24 h and subsequently washing thoroughly with deionized water. The resin was successively treated with 5% HCl and 4% NaOH solutions to remove potential monomers and porogenic agents trapped inside the pores during the synthesis process.

The crude extract was centrifuged at 12000 rpm for 30 min and filtered using a 0.45 μM filter. Subsequently, the extract was purified using a polystyrene column (Ø: 10 mm; h: 300 mm) packed with XDA-7 resin. For polyphenol adsorption, 3.2 mg/mL crude extract was passed through the resin at a flow rate of 1 mL/min. The loaded resin was washed with 3 BV deionized water prior to desorption. Desorption of phenolic compounds from the loaded XDA-7 macroporous resin was performed using 60% (v/v) ethanol solutions at a rate of 2 mL/min. The eluent was collected and concentrated under reduced pressure in a rotary evaporator. Finally, the purified extracts were stored at − 80 °C until further analysis.

The total polyphenol content was determined using the colorimetric Folin–Ciocalteu method with some modifications [15]. Gallic acid was used as a standard phenolic compound. One milliliter of extract solution contained 1 mg extract diluted with distilled water. Five milliliter of Folin-Ciocalteu reagent (10%) was added and the content was mixed thoroughly. After 3–8 min, 4 mL of 7.5% Na_2_CO_3_ was added and the mixture was allowed to stand for 1 h with occasional shaking. The absorbance was measured at 765 nm.

### Cell culture and treatment

Murine L929 fibroblasts were grown in Dulbecco’s modified eagle medium supplemented with 10% (v/v) heat-inactivated fetal bovine serum (FBS) and 1% (v/v) penicillin/streptomycin at 37 °C and 5% CO_2_. Depending on the experiment, the cells were seeded in 96-well plates (1 × 10^5^ cells/mL), 24-well plates (1 × 10^5^ cells/mL), 6-well plates (1.5 × 10^5^ cells/mL), or 60 mm culture dishes (2 × 10^5^ cells/mL), and maintained in a tissue culture incubator overnight to allow adherent monolayer formation. After serum starvation, by culturing in a medium containing 3% FBS for 12 h, the cells were treated with different concentrations of STPE (0–1000 μg/mL) for 24 h. The culture medium was replaced with a thin layer of fresh Hank’s Balanced Salt Solution (HBSS) (Hyclone, Logan, UT, USA) and cells were irradiated with the appropriate UVB dose using a UV spectral radiometer (VLX-3 W, Vilber Lourmat, France). HBSS was subsequently replaced with culture medium cotaining 3% FBS and incubated until analysis.

### Cell viability assay

Cell viability was evaluated using 3-(4,5-dimethylthiazol-2-yl)-5-(3-carboxymethoxyphenyl)-2-(4-sulfophenyl)-2H-tetrazolium (MTS) using the CellTiter 96 Aqueous Non-Radioactive Cell Proliferation assay (Promega, Madison, WI, USA) according to the manufacturer’s instructions. Briefly, L929 cells were seeded in 96-well plates at a density of 10^4^ cells/well, and allowed to attach overnight. Cells were starved by culturing in a medium containing 3% FBS for 12 h. For the cytotoxicity profile evaluation, the cells were treated for 24 h with different concentrations of STPE. For the selection of the exposed dose, the cells were exposed to different doses of UVB (10, 20, 40, 80, 160, and 320 mJ/cm^2^) and incubated for 24 h. For the protective evaluation under UVB rays, the cells were treated for 24 h with STPE, washed twice with HBSS, exposed to radiation, and incubated for a further 24 h. After the respective treatment conditions, 20 μL of MTS solution was added to wells for another 45 min, and the absorbance at 490 nm was measured with a microplate reader (Tecan, Männedorf, Switzerland). At least three independent experiments were performed.

### ROS production

The intracellular accumulation of ROS was detected by 2,7-dichlorodihydrofluorescein diacetate (H_2_DCF-DA) (D6883, Sigma-Aldrich, St. Louis, MO, USA). The cells were treated with STPE for 24 h and then incubated with 20 μM H_2_DCF-DA for 30 min at 37 °C. Subsequently, the cells were washed three times with HBSS and exposed to 100 mJ/cm^2^ UVB. Fluorescence was measured at 488/525 nm 1 h after UVB exposure using Tecan Infinite M200 PRO (Tecan).

### Cellular antioxidant enzyme activity and lipid peroxidation

L929 cells were cultured in 60 mm tissue culture dishes at 1.6 × 10^6^ cells/dish. After treatment with different concentrations of STPE (0, 10, 20, and 40 μg/mL) for 24 h, the cells were exposed to 20 mJ/cm^2^ UVB and incubated for a further 24 h. The cells were collected and resuspended in 400 μL of phosphate-buffered saline (PBS). The cells were disrupted using TissueLyser II (Qiagen, Hilden, Germany) and superoxide dismutase (SOD) and catalase (CAT) within the lysates were measured with appropriate assay kits (Jiancheng Bioengineering Institute, Nanjing, China) according to the manufacturer’s instructions. Intracellular lipid peroxidation was evaluated by measuring malondialdehyde (MDA) production using a commercial kit (Jiancheng Bioengineering Institute, Nanjing, China).

### RNA isolation, reverse transcription, and quantitative PCR (qPCR) analysis

The total RNA sample was collected and extracted using an RNAprep Pure kit (Tiangen Biotech, Beijing, China) 24 h after the end of UVB exposure at 10 mJ/cm^2^. Reverse transcription was performed with 1 μg of total RNA using a PrimeScript RT Master Mix (Takara Bio, Otsu, Japan), and real-time qPCR was performed using a FastStart Universal SYBRGreen Master kit (Roche, Basel, Switzerland). The primers used for amplification of the target genes (inflammation-related genes, such as TNF-α, IL-1α, and IL-6) and the reference gene (succinate dehydrogenase complex flavoprotein subunit A, SDHA) are listed in Table 1. Relative quantification of target gene expression levels was performed using the 2^−ΔΔCt^ method.

**Table 1:** Primers used for real-time qPCR

Gene	Primer sequence (5′ → 3′)	GenBank accession No.	Reference
*SDHA*	GGAACACTCCAAAAACAGACCT	NM_023281	–
	CCACCACTGGGTATTGAGTAGAA		
*IL-1α*	TCTATGATGCAAGCTATGGCTCA	NM_010554	–
	CGGCTCTCCTTGAAGGTGA		
*IL-6*	CCCAATTTCCAATGCTCTCC	NM_031168	[16]
	TCCACAAACTGATATGCTTAGG		
*TNF-α*	ACAAGGCTGCCCCGACTAC	NM_013693	
	TGGAAGACTCCTCCCAGGTATATG		

### Enzyme-linked immunosorbent assay (ELISA)

The cell culture supernatant was collected and concentrated 20 times in an ultrafilter tube prior to cytokine measurement by ELISA, according to the manufacturer’s instructions. The ELISA kits for TNF-α, IL-6, and IL-1α were purchased from Novus Biologicals (Littleton, CO, USA) or ProteinTech (Wuhan, China). Absorbances were determined at 450 nm using a microplate spectrophotometer reader (Tecan), and the results are expressed as picograms (pg) of each cytokine/mL.

### Western blotting

For NF-κB nuclear translocation analysis, after 24 h of UVB irradiation, the separation of cytosolic and nuclear components of L929 cells was performed using NE-PER nuclear and cytoplasmic extraction reagents (Thermo Fisher Scientific), according to the manufacturer’s instructions. The amount of protein was estimated using the PierceTM BCA Protein Assay Kit (Thermo Fisher Scientific). Afterward, equal amounts of protein were collected and heated at 100 °C for 5 min in a loading buffer. The proteins were electrophoresed on a 12% SDS-polyacrylamide gel and transferred to a polyvinylidene fluoride membrane (Pall Corporation, NY, USA) in a transfer buffer (25 mM Tris base, 250 mM glycine, 20% methanol). The membranes were blocked with 5% skim milk (BD Difco, NJ, USA) in PBS containing 0.05% Tween 20 buffer overnight at 4 °C and incubated with primary antibodies (NF-κB p65 (D14E12) XP Rabbit mAb, p-NF-κB p65 (Ser536) (93H1) rabbit mAb, anti-IκBα antibody, anti-SDHA antibody, and anti-Lamin B antibody) for 2 h at room temperature. Membranes were washed and incubated with horseradish peroxidase-conjugated goat anti-rabbit IgG secondary antibody for 1 h at room temperature. The antigen-antibody complex was detected by chemiluminescence using ECL detection reagent (Advansta, CA, USA) and analyzed using the ChemiDoc® XRS+ Imaging System (Tanon-5200 s, Tanon, Shanghai, China) (Additional file 1).

### Hoechst 33342 staining

The intensity of nuclear condensation was examined using the cell-permeable DNA dye Hoechst 33342. The probe (2 μg/mL) was added after 24 h of UVB exposure, and cells were incubated for 20 min at 37 °C and washed twice with PBS. The cells were then visualized under a fluorescence microscope (DMI8, Leica, Wetzlar, German).

### Effects of STPE on zebrafish growth

Embryos of AB wild-type zebrafish developed at 2 days post-fertilization (dpf) were collected and randomly divided into 30 embryos per experimental group. Embryos were treated with STPE at concentrations of 5, 10, 25, 100, 250, 500, 1000, and 2000 μg/mL for 24 h, and exposed to UVB generated by an ultraviolet light therapy instrument (KN-4006, Kernel Medical Equipment, Xuzhou, China) at 3 dpf. For UVB exposure, each group was exposed five times separated by 30 min intervals. Each exposure delivered 8100 mJ/cm^2^ (9 mW/cm^2^, 15 min) of energy. After UVB exposure, all embryos were cultivated in 6-well cell culture plates and the survival rates were counted at 5 dpf. The study was carried out in compliance with the ARRIVE (Animal Research: Reporting of In Vivo Experiments) guidelines.

### Repairing effect of STPE on caudal fin damage in UVB-irradiated zebrafish

Embryos developed at 2 dpf were collected and randomly divided into 30 embryos per experimental group. Embryos were treated with different concentrations of STPE (0.56, 1.67, and 5 μg/mL) or epigallocatechin gallate (EGCG; 30 μg/mL) for 24 h. After UVB exposure at 3 dpf, all embryos were cultivated in 6-well cell culture plates at 28 °C until 5 dpf. The culture medium was changed daily throughout the experiment. Ten zebrafish were randomly selected from each group to observe their tails under a dissecting microscope (SZX7, Olympus, Tokyo, Japan). Images were taken and analyzed using an image processing software (NIS-Elements D 4.30.00) to calculate the area of the caudal fin of the larva. The caudal fin area was used to evaluate the effect of STPE on the repair of UV irradiation-induced skin damage in zebrafish. Skin damage repair of zebrafish was calculated using the following formula: $\mathrm{Repairing}\ \mathrm{effect}\ \left(\%\right)=\frac{{\mathrm{S}}_{\mathrm{S}\mathrm{TPE}}-{\mathrm{S}}_{\mathrm{UVB}}}{{\mathrm{S}}_{\mathrm{UVB}}}\times 100\%$. The study was carried out in compliance with the ARRIVE guidelines.

### Effect of STPE on ROS scavenging in UVB-irradiated zebrafish

Embryos developed at 2 dpf were collected and randomly divided into 30 embryos per experimental group. Embryos were treated with different concentrations of STPE (0.8, 1.2, and 1.6 μg/mL) or EGCG (30 μg/mL) for 24 h. After 2 h of UVB exposure at 3 dpf, embryos were incubated with 500 ng/mL CM-H_2_DCF-DA (Invitrogen, Waltham, USA) for 20 h. The embryos were subsequently placed in a 96-well blackboard (1 per well, 8 per group). The fluorescence intensity of the embryo (FI) was measured at excitation/emission = 485/535 nm (Tecan, Austria). The effect of STPE on oxygen radical scavenging in zebrafish was calculated as follows: $\mathrm{ROS}\ \mathrm{scavenging}\ \mathrm{rate}\ \left(\%\right)=\frac{\left({\mathrm{FI}}_{\mathrm{UVB}}-{\mathrm{FI}}_{\mathrm{Blank}}\right)-\left({\mathrm{FI}}_{\mathrm{STPE}+\mathrm{UVB}}-{\mathrm{FI}}_{\mathrm{Blank}}\right)}{{\mathrm{FI}}_{\mathrm{UVB}}-{\mathrm{FI}}_{\mathrm{Blank}}}\times 100\%$. The study was carried out in compliance with the ARRIVE guidelines.

### HPLC-MS/MS analyses

STPE was analyzed on a Thermo Ultimate 3000 LC system coupled with a Thermo Q Exactive HF system (Thermo Fisher Scientific, Waltham, USA). Chromatographic analyses were carried out on Zorbax Eclipse C18 (2.1 × 100 mm,1.8 μm, Agilent Technologies). The mobile phase consisted of two solvents: 0.1% formic acid in water (A) and acetonitrile (B). Elution was performed with linear gradient elution as follows: 0–2 min, 5% B; 2–6 min, 5–30% B; 6–7 min, 30% B; 7–12 min, 30–78% B; 12–14 min, 78% B; 14–17 min, 78–95% B; 17–20 min, 95% B; 20–21 min, 95–5% B; 21–25 min, 5% B. The flow rate was 0.3 mL/min and the injection volume was 2 μL.

The mass spectrometer was operated in both positive and negative ion modes and full MS (m/z 100–1500)/ data-dependent MS^2^ (dd-MS^2^, TopN = 10) mode. The mass parameters were as follows: spray voltage 3.5 kV; capillary temperature 330 °C; sheath gas (N_2_) flow rate 45 arb; auxiliary gas (N_2_) flow rate 15 arb; probe heater temperature 325 °C; and S-Lens RF level 55%. Retention time correction, peak identification, peak extraction, etc. were performed using Compound Discoverer 3.2. The identification of individual phenolic compounds was based on their retention time, mass spectrometry, and their mass-to-charge (m/z) ratio as compared with local brown algae polyphenol database constructed based on previous literature.

## Results

### STPE reverses the UVB-induced decrease in cell viability

*S. thunbergii* was extracted twice at 70 °C with 40% anhydrous ethanol, and then purified with the macroporous adsorbent resin XDA-7 to obtain STPE. The total polyphenol content of STPE was 72% as determined using the colorimetric Folin–Ciocalteu method.

**Fig. 1:**
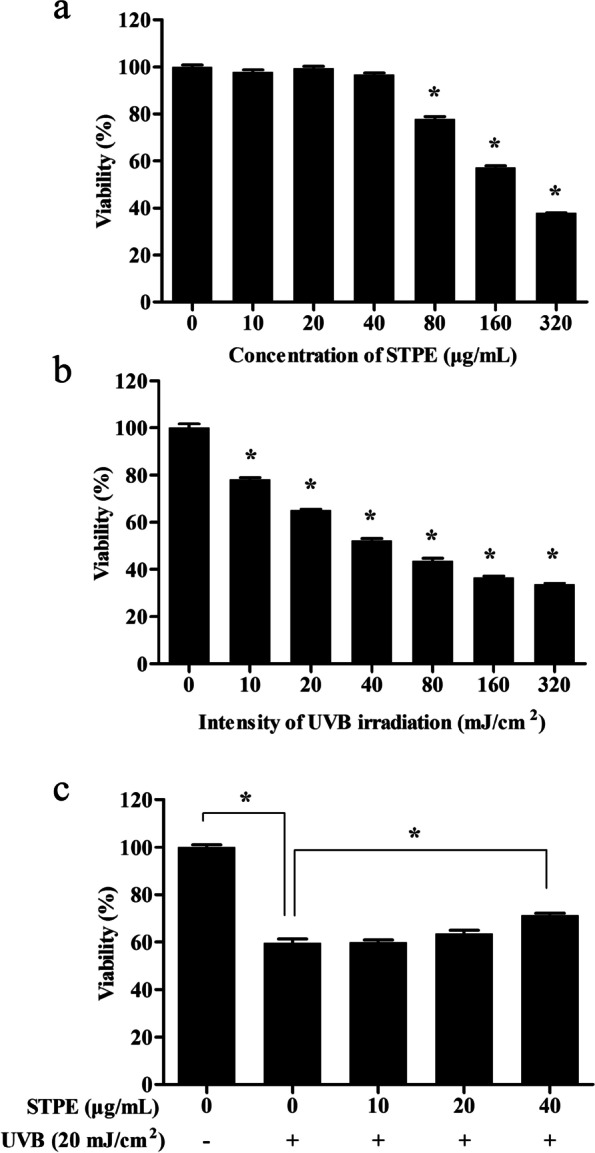
STPE reverses the UVB-induced decrease in cell viability. The effects of (**a**) STPE and (**b**) UVB at different concentrations or dose on L929 cell viability, and (**c**) the protective effects of STPE on L929 cell damage by UVB (20 mJ/cm^2^). The error bars refer to the standard deviations obtained from six sample analyses. The data were analyzed by one-way ANOVA followed by LSD post-hoc test. Means with “*” differ significantly (*p* < 0.05)

To determine STPE cytotoxicity, L929 cells were treated with various concentrations of STPE (0–320 μg/mL) for 24 h. STPE at concentrations of 10–40 μg/mL had no effect on cell viability compared to the untreated group (*p* > 0.05; Fig. 1a), although cytotoxicity was observed at 80 μg/mL (*p* < 0.05). Based on this, 10–40 μg/mL STPE was used in subsequent assays. Cells were treated with various doses of UVB (0–320 mJ/cm^2^) and cultured for 24 h to determine the appropriate irradiation dose to induce photodamage. UVB reduced cell viability in a concentration-dependent manner (Fig. 1b); 20 mJ/cm^2^ UVB decreased cell viability by approximately 50% and was selected as the UVB dose in subsequent experiments. Cell viability was reduced to 60% following exposure to UVB alone compared with that of the experimental control (Fig. 1c). However, pretreatment of cells with STPE (10–40 μg/mL) reversed this effect in a concentration-dependent manner, and was significantly different at 40 μg/mL from that of the UVB model group.

### STPE inhibits oxidative stress and lipid peroxidation

**Fig. 2:**
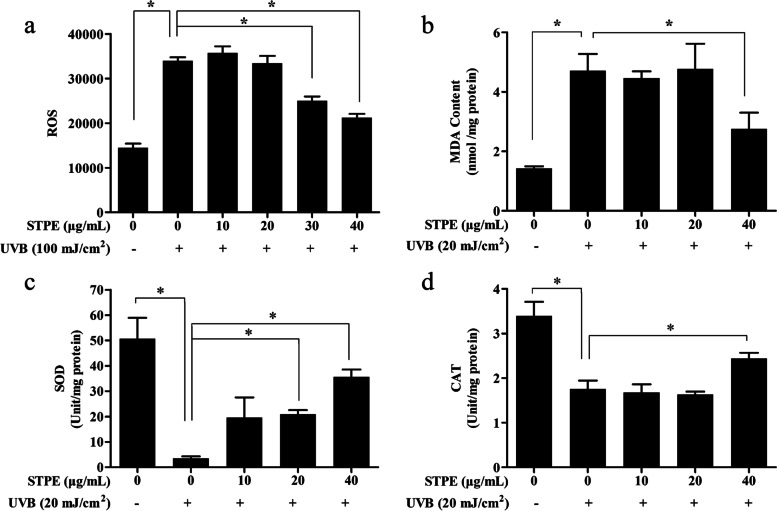
STPE inhibits UVB-induced oxidative stress and lipid peroxidation. Effects of STPE on (**a**) ROS and (**b**) MDA content as well as (**c**) SOD and (**d**) CAT activities in L929 cells. Means with “*” differ significantly (*p* < 0.05)

The intracellular ROS scavenging effects of STPE induced by UVB were assessed using H_2_DCF-DA oxidant-sensitive fluorescent probes. The scavenging effect of STPE on the intracellular ROS generated by UVB exposure was measured using a microplate reader (Fig. 2a). The ROS level in UVB-exposed cells increased 2.35-fold compared with that in control cells. However, 24 h pretreatment of cells with increasing concentrations of STPE resulted in a concentration-dependent reduction of intracellular ROS.

SOD and CAT are important antioxidant enzymes that protect tissues from oxidative damage. MDA production is used to evaluate intracellular lipid peroxidation owing to oxidative stress. The protective mechanisms of STPE were evaluated by measuring the levels of SOD, CAT, and MDA. Compared with those in the control group, SOD levels decreased following UVB exposure; however, this was abrogated by STPE pretreatment at 20 and 40 μg/mL (Fig. 2c). CAT activity was elevated after STPE treatment at 40 μg/mL (Fig. 2d). The increase in MDA content (Fig. 2b) induced by UVB (*p* < 0.05) was reversed by pretreatment with 40 μg/mL STPE (*p* < 0.05).

### STPE alleviates UVB-induced inflammation in L929 cells

**Fig. 3:**
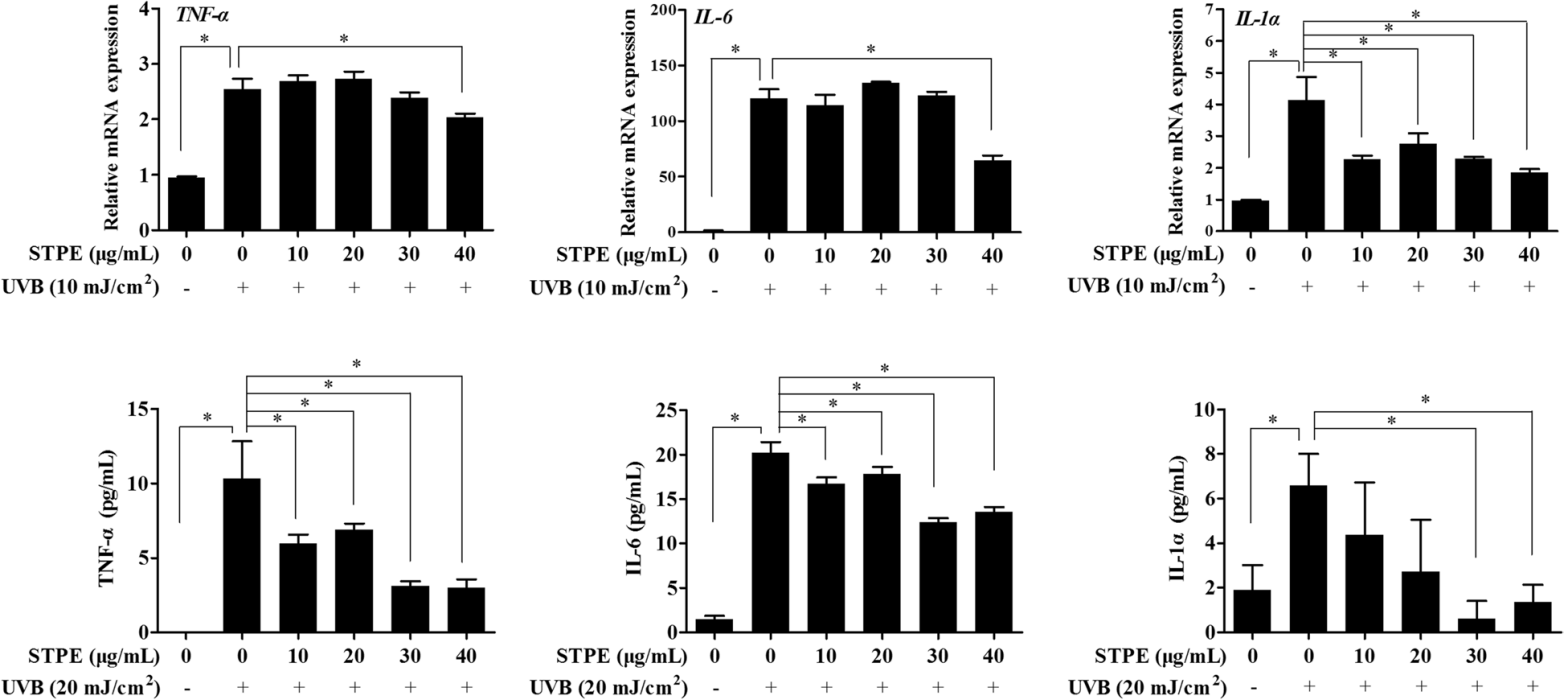
STPE inhibits UVB-induced pro-inflammatory cytokine mRNA expression and secreted production in L929 cells. Cells were pretreated with different concentrations of STPE (0, 10, 20, and 40 μg/mL) for 24 h. **a** The mRNA expression of TNF-α, IL-6, and IL-1α in L929 cells was measured by qPCR at 24 h after the end of exposure to 10 mJ/cm^2^ UVB. **b** Cell culture supernatants were collected to measure cytokine production at 24 h after the UVB exposure (20 mJ/cm^2^). Bars represent means ± SEM of four samples per group. The data were analyzed by one-way ANOVA followed by LSD post-hoc test. Means with “*” differ significantly (*p* < 0.05)

To evaluate the effects of STPE on inflammatory cytokine production in UVB-exposed L929 cells, the mRNA and protein expression levels were measured by real-time qPCR (Fig. 3a) and ELISA (Fig. 3b), respectively. UVB exposure stimulated the expression of *TNF-α*, *IL-1α*, and *IL-6* mRNA compared with control cells. However, treatment with 40 μg/mL STPE decreased *TNF-α* and *IL-6* mRNA expression levels. STPE reduced UVB-induced TNF-α, IL-6, and IL-1α protein production in a dose-dependent manner.

### STPE inhibits NF-κB activation in UVB-induced L929 cells

**Fig. 4:**
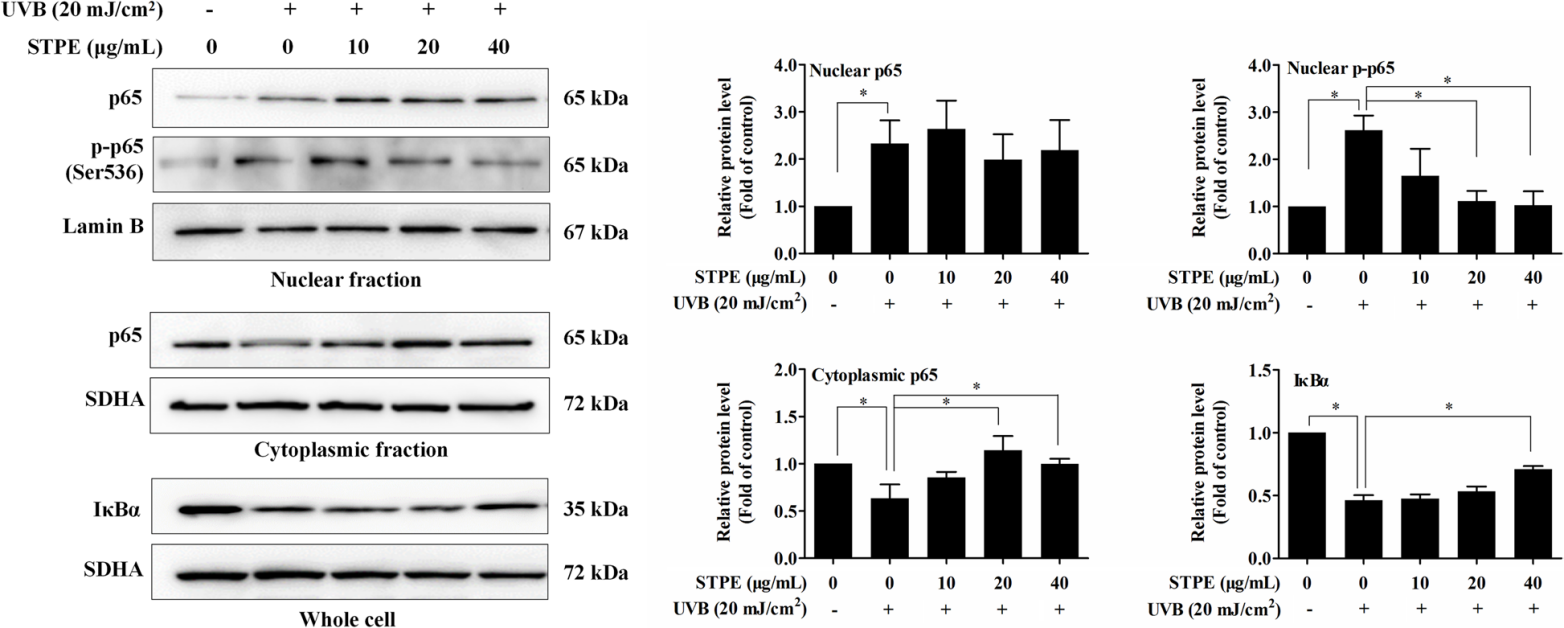
Effects of STPE on NF-κB translocation and I-κB𝛼 expression in UVB-induced L929 cells. Western blot analysis showing the effect of STPE treatment on the expression of p65 and p-p65 in nuclear extracts, p65 in cytoplasmic extract, and I-κB𝛼 in the whole cell. Cytosolic proteins were normalized with SDHA and nuclear proteins were normalized with laminin B. The cells were grown in 6-well cell culture plates and treated with various concentrations of STPE for 24 h before UVB (20 mJ/cm^2^) treatment. After incubation for 24 h, cell lysates were collected and subjected to western blot analysis using antibodies specific for p65, p-p65, and I-κB𝛼. Intensity quantification of protein bands were analyzed by Quantity One software (Bio-Rad). Bars represent means ± SEM of three samples per group. The data were analyzed by one-way ANOVA followed by LSD post-hoc test. Means with “*” differ significantly (*p* < 0.05)

NF-κB plays an important role in immune responses, and dysregulation of NF-κB is associated with various diseases such as cancer, inflammation, and aging [17]. UVB increased both the protein levels of NF-κB p65 and p-p65 (Ser536) in the nucleus, whereas treatment with STPE decreased the nuclear p-p65 (Ser536) protein levels in a dose-dependent manner (Fig. 4). The protein expression levels of p65 did not decrease in the nucleus after STPE incubation. Additionally, STPE pretreatment significantly increased NF-κB (p65) expression in the cytoplasm. IκBs are upstream regulators of the NF-κB signaling pathway. In cells that are not stimulated, NF-κB dimers are present in an inactive state by binding to IκBs. IκBs are degraded by ubiquitination; thus, NF-κB is translocated from the cytoplasm to the nucleus, causing an inflammatory response following UVB exposure [18]. Our results showed that treatment with STPE at 40 μg/mL significantly enhanced IκBα expression. The increased protein levels of cytoplasmic NF-κB (p65) and IκBα indicated that STPE prevents NF-κB from entering the nucleus.

### STPE attenuates DNA condensation in UVB-treated L929 cells

**Fig. 5:**
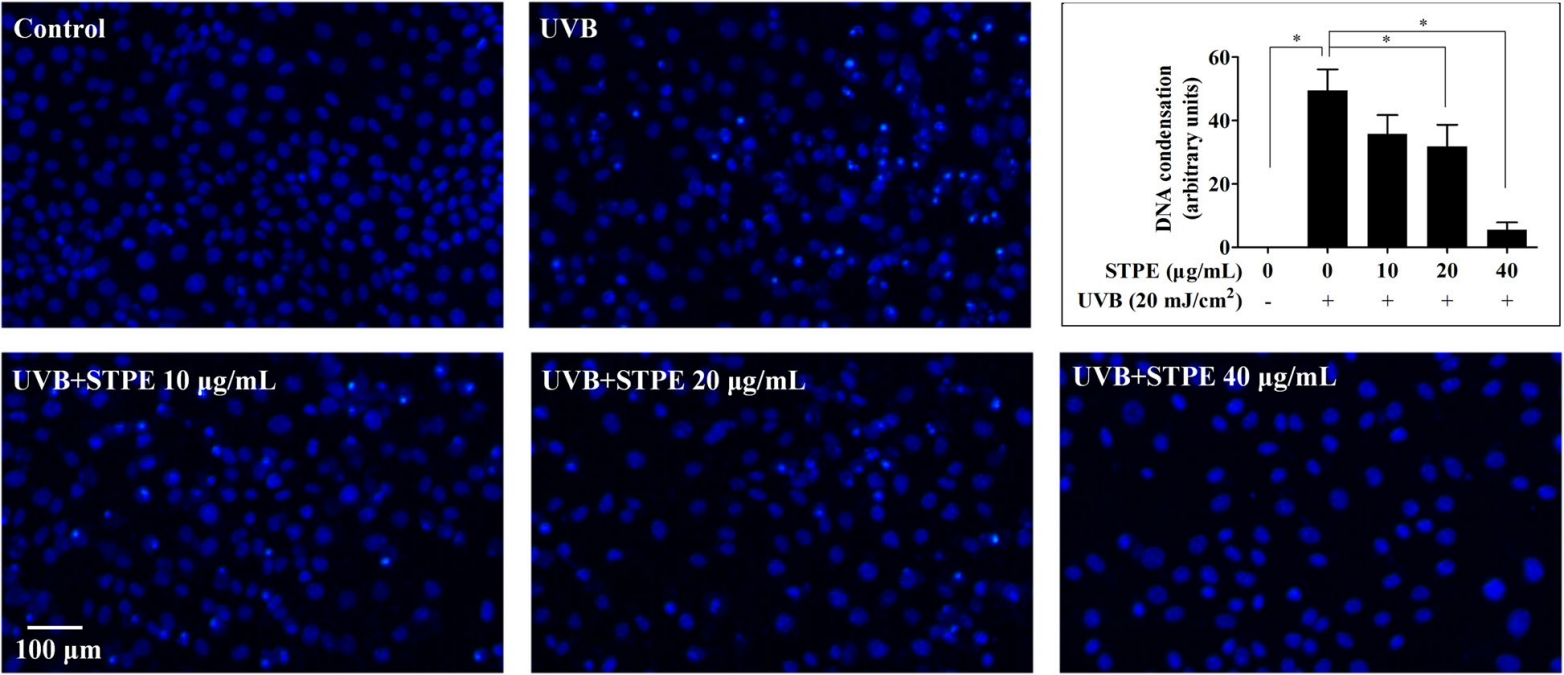
Evaluation of DNA protection in UVB-irradiated L929 fibroblasts pretreated with STPE. Apoptotic body formation was observed under a fluorescence microscope following Hoechst 33342 staining. Bars represent means ± SEM of four samples per group. The data were analyzed by one-way ANOVA followed by LSD post-hoc test. Means with “*” differ significantly (*p* < 0.05)

To evaluate nuclear alterations, DNA condensation was analyzed and visualized by fluorescence microscopy (Fig. 5). The fluorescent probe, Hoechst, was used to evaluate DNA condensation. Cells with homogeneously stained nuclei were considered viable, whereas cells stained strongly with an intense blue color were indicative of apoptosis [19]. Untreated cells displayed intact nuclei, whereas UVB-irradiated cells exhibited significant nuclear condensation (bright blue staining), suggesting these cells were undergoing the apoptotic process. Pretreatment with 20 and 40 μg/mL STPE significantly reversed DNA condensation compared with the UVB control.

### Repairing effect of STPE on caudal fin damage in UVB-irradiated zebrafish

**Fig. 6:**
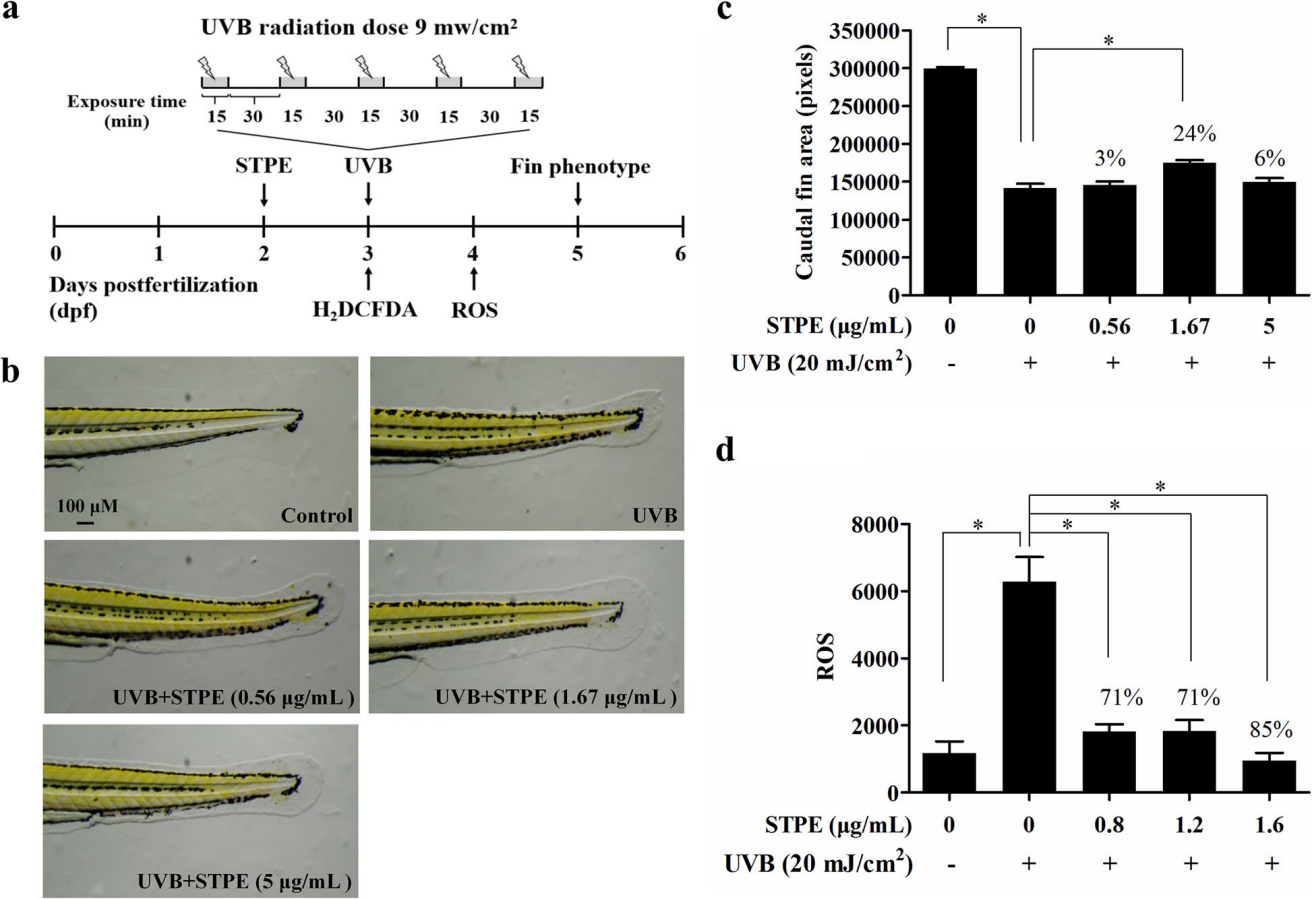
Evaluation of photoprotective effect of STPE on UVB-irradiated zebrafish. Zebrafish embryos at 2 days post-fertilization (dpf) were used. **a** Schematic representation of the experimental protocols performed in this study. **b**, **c** UVB-induced malformed fin phenotypes can be attenuated by STPE. **d** Inhibitory effect of STPE on UVB-induced ROS generation in zebrafish. Results were analyzed using one-way ANOVA with post-hoc Dunnett’s t-test. Differences with a *p* value of less than 0.05 were considered statistically significant and denoted by “*”

Zebrafish have been widely used in recent years as an in vivo oxidative stress model to study the protective effects of UV radiation. Schematic representation of the experimental protocols was shown in Fig. 6a. This study first explored the effects of STPE on the growth of wild-type AB strain zebrafish, thereby providing data on the maximum safe STPE concentration for subsequent experiments (Additional file 1). STPE induced high mortality at concentrations of 10 μg/mL and above after UV irradiation. No significant abnormalities were observed at 5 μg/mL STPE; therefore, this was the maximum experimental concentration used in the zebrafish UV damage model.

The potential of STPE to repair skin damage in zebrafish exposed to UVB radiation is shown in Fig. 6b and c. Using a deconvolution microscope, the caudal fin of zebrafish displayed a rough and crinkled phenotype after exposure to UV radiation. Treatment of zebrafish with 30 μg/mL of EGCG showed 182,115 ± 8402 pixels in the tail fin area of zebrafish, compared to only 141,544 ± 5864 pixels in the tail fin area of the control zebrafish model. The effect of tea polyphenols on the repair of zebrafish skin damage was 29%, indicating that the positive control treatment significantly repaired zebrafish skin damage. Treatment of zebrafish with 0.56, 1.67, and 5 μg/mL of STPE resulted in a caudal fin area of 145,518 ± 4608 (*p* > 0.05), 174,965 ± 3632 (*p* < 0.001), and 149,357 ± 5283 (*p* > 0.05) pixels, respectively, compared with that of the control model; the effects on skin damage repair were 3, 24, and 6%, respectively. Collectively, STPE showed a significant restorative effect on zebrafish skin damage.

### ROS scavenging effect of STPE on UVB-radiated zebrafish

The effects of STPE on UVB-mediated ROS induction in zebrafish are shown in Fig. 6d. The fluorescence value of the UVB group was 6271 ± 264 (*p* < 0.001), which was higher than that of the normal controls (1169 ± 124), indicating successful model establishment. The fluorescence values of the 30 μg/mL EGCG-positive control group (1122 ± 39) were reduced by 82% (*p* < 0.001) compared with those of the UVB group. The fluorescence values of the STPE group at 0.8, 1.2, and 1.6 μg/mL concentrations were 1818 ± 78, 1834 ± 116, and 950 ± 30, respectively, at which the intracellular ROS accumulation decreased by 71, 71, and 85% (*p* < 0.001), respectively, compared with those of the UVB group. These findings demonstrated that STPE had a significant scavenging effect on ROS in this zebrafish model.

### Phenolic profile of STPE

**Fig. 7:**
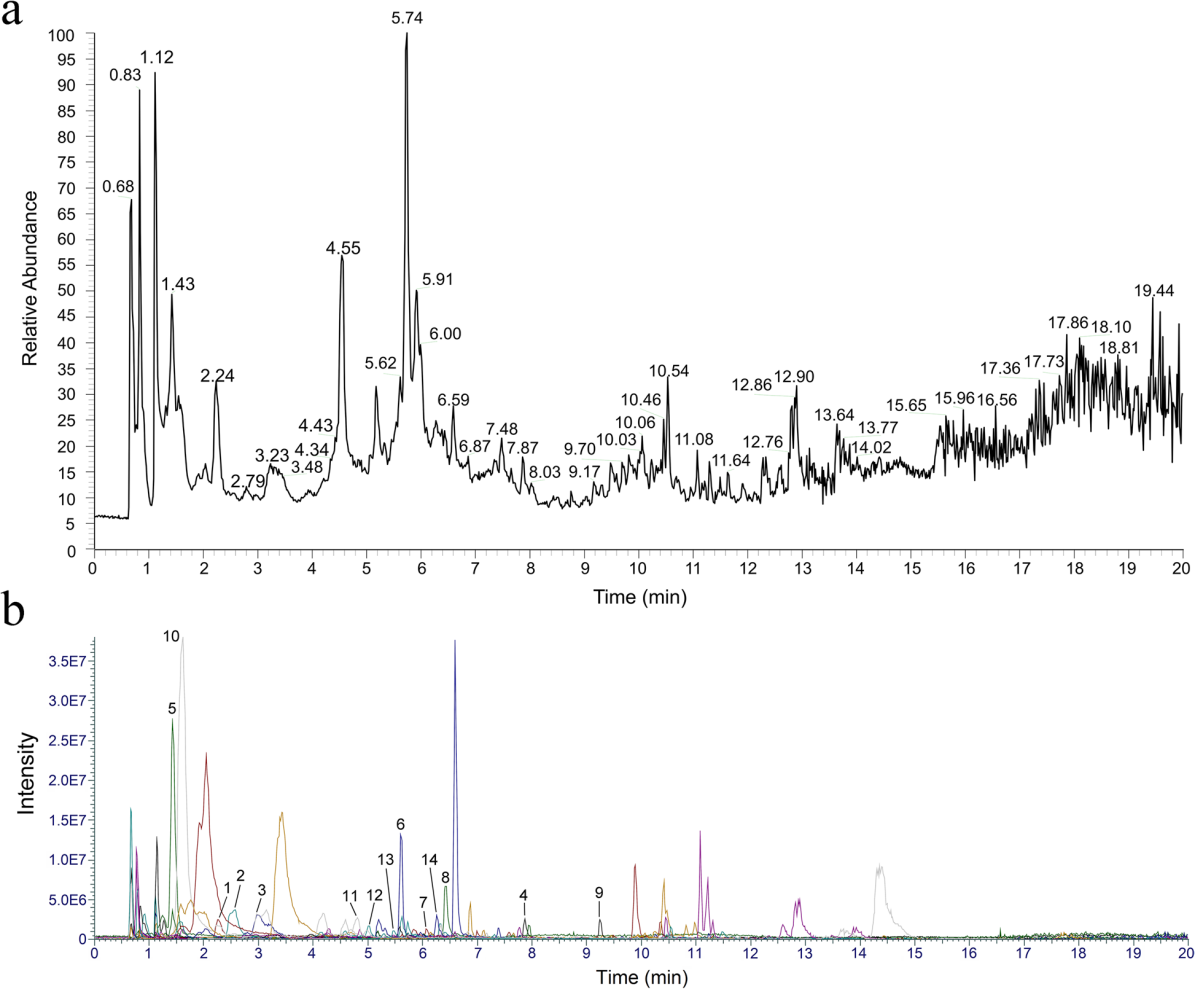
Total ion chromatogram (**a**) and Overlaid extracted ion chromatogram (EIC) acquired in negative ion mode (**b**) for STPE. Peaks refer to Table 2

The total ion chromatogram for the determination of STPE in negative ion mode is shown in Fig. 7a. To extract information about the phenolic compounds, the names and molecular formulae of 144 phenolic compounds were collected from professional databases (Pubchem, ChemSpider, Scifinder and Huaxuejia) and references related to the phenolic extracts of brown algae, and their exact relative molecular masses were calculated. 14 phenolic compounds identified from STPE, which included 4 Benzene and substituted derivatives, 1 Cinnamic acids and derivatives, 4 Flavonoids and 5 phlorotannins (Table 2). The extracted ion chromatograms are shown in Fig. 7b.

**Table 2:** Peak assignments of brown algae phenolic compounds detected in STPE

No.	RT (min)	Compond	Formula	Class	[M-H]^−^ m/z
1	2.27	protocatechuic acid	C_7_H_6_O_4_	Benzene and substituted derivatives	153.01883
2	2.55	difucol	C_12_H_10_O_6_	Benzene and substituted derivatives	249.04079
3	2.99	gallic acid	C_7_H_6_O_5_	Benzene and substituted derivatives	169.01376
4	7.87	4-hydroxybenzoic	C_7_H_6_O_3_	Benzene and substituted derivatives	137.02383
5	1.43	p-Coumaric acid	C_9_H_8_O_3_	Cinnamic acids and derivatives	163.0396
6	5.60	Isoquercitrin	C_21_H_20_O_12_	Flavonoids	463.08957
7	6.07	quercitrin	C_21_H_20_O_11_	Flavonoids	447.09467
8	6.42	isorhamnetin	C_16_H_12_O_7_	Flavonoids	315.05206
9	9.24	catechin	C_15_H_14_O_6_	Flavonoids	289.07248
10	1.62	bifuhalol	C_12_H_10_O_7_	Tannins	265.03595
11	4.81	pentafuhalol A	C_30_H_22_O_17_	Tannins	653.08099
12	5.01	7-hydroxyeckol	C_18_H_12_O_10_	Tannins	387.03696
13	5.48	deshydroxypentafuhalol	C_30_H_22_O_16_	Tannins	637.08289
14	6.33	trifuhalolA	C_18_H_14_O_10_	Tannins	389.05243

## Discussion

UVB radiation causes a series of adverse skin reactions, such as erythema, sunburn, photoaging, and cancer, by altering the signaling pathways related to inflammation and oxidative stress. Therefore, improving UVB-induced inflammation and oxidative stress is a potential tool for preventing and protecting against UVB-induced cellular and tissue photodamage [20]. In this regard, several edible and medicinal plants have been used to mitigate UVB-exposed skin damage. The present study demonstrates the inhibitory effect of *S. thunbergii* against UVB-induced oxidative stress and inflammation, suggesting the potential photoprotective effect of STPE in UVB-irradiated L929 cells in vitro*,* as well as zebrafish in vivo.

L929, a mouse fibroblast cell line, has been used for phototoxicity assessment, and the results were compared with the OECD-approved NIH-3 T3 cell line [12]. The study showed that the L929 cell line, with UVR, was found to be an equally potential in vitro screening test system for the photosensitivity evaluation of drugs/chemicals; thus, it could be used as an in vitro screening test system for phototoxicity assessment. At the same time, the literature also shows that many studies have used L929 fibroblast cell lines as a target of UVB-induced photoaging and photodamage [21–24], which also suggested the suitability of mouse L929 as an in vitro model of photodamage.

UVB induces ROS generation via the BLT2-Nox1-linked pathways, and leads to the depletion of antioxidant defenses either by exhausting their activity or directly damaging proteins [4, 25]. Excessive ROS attack the cell membrane and cellular organelles, resulting in lipid peroxidation and organelle damage, which further induce inflammation and apoptosis in human skin cells [26]. To reduce the negative impact of UV radiation, many marine organisms that photosynthesize have adapted to evolve multiple photoprotective mechanisms against UV radiation [27]. Under UVB radiation, plants alter their phenolic metabolism and provide phenolic compounds to shield against the UVB rays, and act as an antioxidant to decrease UVB-mediated ROS production [28]. In our study, STPE possessed ROS clearance in both L929 cells and zebrafish models. The ROS scavenging ability of brown algae polyphenol in relation to skin protection against UVB have been widely demonstrated. Heo et al. [29] demonstrated for the first time that dieckol extracted from *E. cava* possesses potential inhibitory effects on intracellular oxidative damage induced by UVB radiation. Ko et al. [30] compared the protective effects of extracts from six species of brown seaweed. The results indicated that dieckol from *E. cava* exhibits a higher protective effect against UVB-induced cell damage in HaCaT cells. Moreover, ROS, nitric oxide, and cell death levels in live zebrafish induced by UVB radiation are reduced following the addition of dieckol.

Previous studies have shown that the expression of several enzymes (CAT, SOD, glutathione transferase, nicotinamide adenine dinucleotide (phosphate) quinone oxidoreductase, and hemoxygenase-1) in skin is affected following exposure to UVB radiation [31]. MDA is a marker of lipid peroxidation and cell injury. Thus, we sought to measure the SOD, CAT, and MDA levels in L929 cells to evaluate the antioxidative effects of STPE. The result showed that STPE enhanced SOD and CAT activity and suppressed MDA production in UVB-irradiated L929 cells. This result was similar with that reported by Jang et al. [32], who reported that phlorotannins eckstolonol from *E. cava* reduced photo-oxidative stress and DNA damage by scavenging ROS and enhancing CAT and SOD activities. The ethyl acetate fraction of *Sargassum muticum* increased the protein expression levels of both Cu/Zn SOD and CAT in a time-dependent manner compared with that of UVB-exposed HaCaT cells [33]. Quercitrin, which also isolated from many brown algae, restored CAT expression and GSH/GSSG ratio reduced by UVB exposure, leading to reductions of oxidative DNA damage and apoptosis and protection of the skin from inflammation caused by UVB exposure [34].

With respect to the mechanism of antioxidant enzyme activity restoration, it has been demonstrated that natural sourced antioxidants regulate the signaling pathways related to the expression of antioxidant enzymes, such as Nrf2-Keap1-ARE, NF-κB and MAPK [35]. Nrf2 regulates the antioxidant factors, including quinone oxidoreductase 1, heme Oxygenase-1, SOD and CAT [36]. During activation of the NF-κB pathway, intranuclear p65 negatively regulates the Nrf2 pathway by competing with the transcriptional co-activator CREB-binding protein (CBP)-p300 complex limiting the availability of CBP for Nrf2 complex formation [37]. In our study treatment with STPE significantly decreased the nuclear p-p65 (Ser536) protein levels, which might release more CBP to bind to Nrf2, subsequently restoring the activities of antioxidant enzymes, such as SOD and CAT. Similar results was found with tea polyphenol pretreatment which significantly inhibited the activation of NF-κB and promoted the activities of SOD, CAT, and GSH-Px [38].

Most UVB-associated diseases share inflammation-related characteristics, such as upregulated pro-inflammatory cytokine levels and the activation of transcription factors, including NF-κB [39, 40]. This, in turn, propagates inflammation-induced signals and aggravates skin aging or disease by inducing apoptosis and increasing ROS production [41]. Martinez et al. [4] reported that UVB irradiation increases the production of several pro-inflammatory cytokines, including TNF-α, IL-1β, and IL-6, as well as Th1, Th2, and Th17 cytokines, and anti-inflammatory cytokines (IL-10 and TGF-β) in the skin of hairless mice. Findings from our study confirmed that UVB markedly induced the gene and protein expression of TNF-α, IL-6, and IL-1α in L929 cells, which was effectively suppressed by pretreatment with STPE. Phenolic compounds have been shown to be potentially useful to treat many inflammatory diseases. Brown algae polyphenol treatment inhibits the expression levels of inflammatory mediators (nitric oxide, iNOS, and COX-2) at the transcriptional level in a *Propionibacterium acnes-*induced HaCaT cell model [42] and macrophage-derived chemokine MDC/CCL22 production in an IFN-γ-induced skin inflammation model [43]. The expression of iNOS, COX-2, TNF-α, IL-6, and HMGB-1 is inhibited following phlorotannin treatment in a lipopolysaccharide-induced sepsis model [44]. NF-κB activation has been shown to regulate the production of a variety of pro-inflammatory cytokines. NF-κB is composed of p65 and p50 subunits and is sequestered in the cytoplasm by its inhibitory proteins, I-κBs. NF-κB p65 can be activated and rapidly transported from the cytoplasm to the nucleus during the phosphorylation and degradation of I-κBs. Herein, STPE inhibited the activation of NF-κB in UVB-irradiated L929 cells, which was supported by a decrease in the phosphorylation of the p65 subunit in the nucleus. Moreover, NF-κB (p65) expression in the cytoplasm and IκB expression were significantly increased by STPE, which also indicated the inhibition of NF-κB activation. Our data suggested that STPE inhibits the UVB-induced inflammatory process in L929 cells through the NF-κB signaling pathway. These results are corroborated by previous reports showing that the purified phlorotannins fucofuroeckol-A and 8,8′-bieckol can reduce the production of pro-inflammatory cytokines via the suppression of NF-κB signaling [45, 46]. Diphlorethohydroxycarmalol (DPHC), isolated from the brown algae *I. okamurae*, suppresses intracellular ROS, collagenase, and elastase production and reduces the expression of MMPs and pro-inflammatory cytokines by regulating the NF-κB, AP-1, and MAPKs signaling pathways in UVB-irradiated human dermal fibroblasts [47].

The zebrafish, a small tropical freshwater fish, has become a useful vertebrate model organism owing to its small size, large clutches, transparency, low cost, and physiological similarity to mammals [48]. Previous studies have used zebrafish as a system for evaluating the efficacy of other UVB protective compounds by assessing fin damage and measuring ROS generation [13, 14]. Our results demonstrated that zebrafish fins in the UVB and STPE combined groups were more likely to phenotypically return to normal than fins in the UVB only-treated group. Additionally, STPE significantly reduced ROS production in the UV-exposed zebrafish embryos. The protective effect of phlorotannin extracts on UVB-radiated zebrafish has been previously reported. For example, DPHC isolated from *I. okamurae* confers in vivo protection against photodamage by decreasing cell death, as well as reducing lipid peroxidation and inflammatory responses by decreasing ROS levels in UVB-irradiated zebrafish [47]. The ethyl acetate fraction of 70% ethanol extracts of *Spirogyra* sp. reduces ROS generation in UVB-irradiated zebrafish [49]. Additionally, Guinea et al. [50] showed that the phenolic extracts from *Macrocystis pyrifera* and *Porphyra columbina* exhibit good photoprotective activity in zebrafish embryo models.

Acute and chronic exposure of skin cells to UVB can cause apoptosis [25], characterized by membrane blebbing and nuclear fragmentation. Skin cells exposed to UVB irradiation may respond by activating protective mechanisms or eventually undergo apoptosis [51]. To further study the protective effect of STPE against UVB radiation-induced apoptosis in L929 cells, apoptotic body formation was observed following Hoechst 33342 staining. Our results showed that UVB greatly enhanced DNA condensation; however, this was significantly decreased by STPE pretreatment. The inhibition of apoptosis by brown algae polyphenol has been previously reported. For example, the suppressive effect of fucodiphlorethol G on apoptotic body formation by UVB radiation has been observed using Hoechst 33342 staining methods [8]. Dioxinodehydroeckol, a phlorotannin from *E. cava*, prevents UVB-induced apoptosis in HaCaT cells [52]. Eckol and triphlorethol-A compounds, purified from *E. cava,* exhibit protective effects against UVB radiation-induced cell damage and apoptosis in human keratinocytes [53, 54].

We identified 14 phenolic compounds from STPE, some of which have been reported to protect skin or skin cells from UVB damage. For example, p-coumaric acid has been reported to attenuate UVB-induced release of stratifin from HaCaT cells and indirectly regulate matrix metalloproteinase 1 release from fibroblasts [55]. Isoquercitrin exhibited antioxidant activity and UVB-induced generation of photoaging-related factor inhibition without showing any toxicity [56]. Gallic acid regulates skin photoaging in both fibroblast and mice model [57]. Quercitrin decreased ROS generation and restored the levels of two major antioxidant enzymes, leading to reductions in UVB-induced oxidative damage [34]. Based on previous reports, the photoprotective activity of STPE may be the result of the combined effect of multiple phenolic compounds.

## Conclusions

**Fig. 8:**
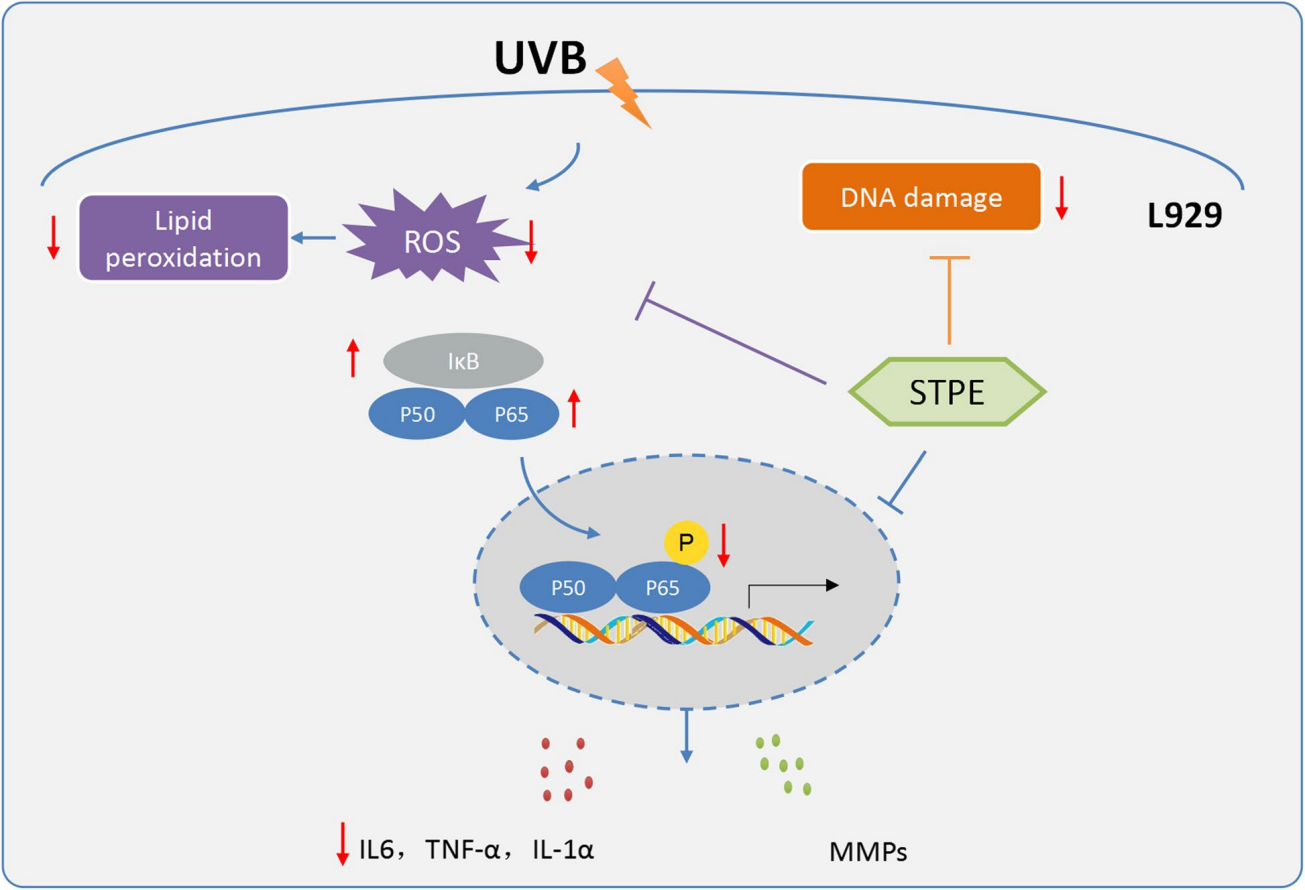
Schematic diagram showing the effects of STPE on UVB induced photodamage

In the present study, we isolated phenolic-rich extract from *S. thunbergii*, and evaluated its protective effects against UVB-induced photodamage, both in vitro using L929 cells and in vivo using zebrafish models. Our data suggest that the protective effects against UVB are mediated through the antioxidant, anti-inflammatory, and anti-apoptotic capacity of STPE by regulating NF-κB signaling pathways (Fig. 8). This study helps to expand our understanding of the protective activity of STPE on skin. However, the pure phenolic compound of STPE with photoprotective activity was not obtained. We will continue to work on obtaining a highly purified and stable STPE molecule for further application as a potential protective agent to prevent skin damage caused by UVB in pharmaceutical and cosmeceutical industries.

## Supplementary Information

**Additional file 1.**

## Data Availability

The gene analyzed during the current study are available from Genbank (https://www.ncbi.nlm.nih.gov/genbank/). The accession numbers are provided as Table 1. Other datasets used and/or analyzed during the current study are available from the corresponding author on reasonable request.

## Abbreviations

CAT: Catalase
dpf: Days post-fertilization
EGCG: Epigallocatechin gallate
ELISA: Enzyme-linked immunosorbent assay
FBS: Fetal bovine serum
FI: Fluorescence intensity of the embryo
HBSS: Hank’s Balanced Salt Solution
H_2_DCF-DA: 2,7-dichlorodihydrofluorescein diacetate
MDA: Malondialdehyde
MTS: 3-(4,5-dimethylthiazol-2-yl)-5-(3-carboxymethoxyphenyl)-2-(4-sulfophenyl)-2H-tetrazolium
NF-κB: Nuclear Factor KappaB
PBS: Phosphate-buffered saline
qPCR: Quantitative PCR
ROS: Reactive oxygen species
SDHA: Succinate dehydrogenase complex flavoprotein subunit A
SOD: Superoxide dismutase
STPE: *S. thunbergii* phenolic-rich extract
UV: Ultraviolet
UVB: Ultraviolet B
