# Supplementary material for: Photoprotective effects of Sargassum thunbergii on ultraviolet B-induced mouse L929 fibroblasts and zebrafish
Source: BMC Complement Med Ther. 2022 May 21;22:144. doi: 10.1186/s12906-022-03609-x (PMC9123674; doi:10.1186/s12906-022-03609-x)

Table S1 Effects of STPE and UVB on zebrafish growth

	STPE Concentration（μg/mL）	Death rate（%）	Toxicological phenotyping
Control	-	0	No significant abnormalities were observed
UVB	-	0	No significant abnormalities were observed
STPE+UVB	5	0	No significant abnormalities were observed
	10	56.7%	No significant abnormalities were observed
	25	100%	-
	100	100%	-
	250	100%	-
	500	100%	-
	1000	100%	-
	2000	100%	-

Fig. S1 Original images from the western blot data presented in main figures

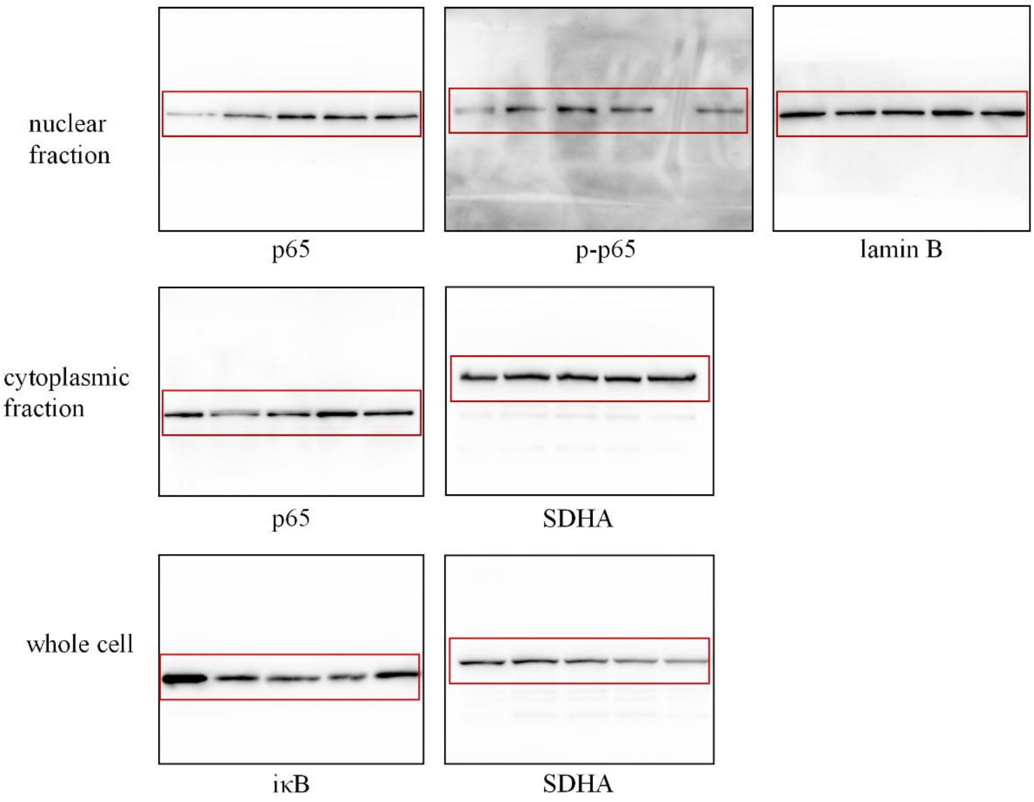

Fig. S2 The imaging of western blot with multiple exposure time

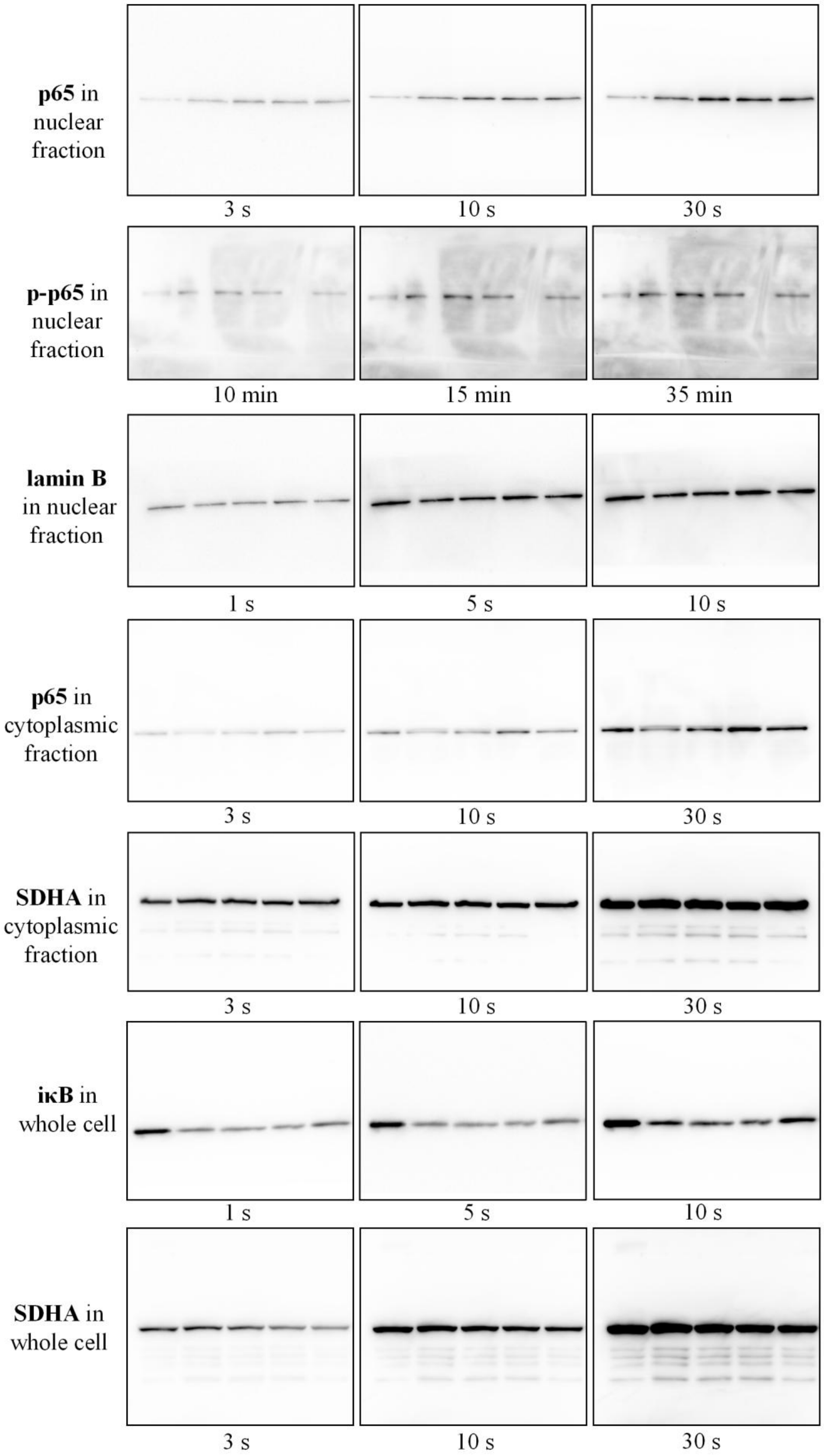

Supplement: Supplementary file 1 — Additional file 1. [file 12906_2022_3609_MOESM1_ESM.pdf]
